# Maillard-type neoallergens present in processed soy extract may cause an allergic reaction in soy allergic patients

**DOI:** 10.1186/2045-7022-5-S3-P21

**Published:** 2015-03-30

**Authors:** Malgorzata Teodorowicz, A P H Jansen, M H W M Roovers, J Ruinemans-Koerts, H J Wichers, H F J Savelkoul

**Affiliations:** 1 Department Cell Biology and Immunology, Wageningen University and Research Centre, Wageningen, The Netherlands; 2 Department of Otorhinolaryngology, Radboud University Nijmegen Medical Centre, Nijmegen, The Netherlands; 3 Department of Allergology, St. Elisabeth Hospital, Tilburg, The Netherlands; 4 Department of Clinical Chemistry and Haematology, Rijnstate Hospital, Arnhem, The Netherlands; 5 Food and Biobased Research, Wageningen University and Research Centre, Wageningen, The Netherlands

## Background

Soybean is an important food allergen and it is often incorporated in complex formulated foods. Thermal processing induces conformational changes of food allergens and accelerates its interaction with carbohydrates. This reaction is called Maillard reaction (MR) and occurs always during food processing. It has been shown to influence allergenic potential of several proteins.

## Objective

The aim of the study was to establish the effect of processing conditions on allergenic potential of soy protein extract containing the major soy allergens Gly m 5 and 6.

## Material and methods

Clinically characterized soy allergic patients (n=15) with specific IgE against Gly m 5 and 6 were selected for the study. Here we show the complete data obtained for seven patients. Five chemically characterized soy protein extracts (SPE) were tested: raw SPE, SPE heated (121^o^C) for 10 or 30 minutes with glucose and SPE heated (121^o^C) for 10 or 30 minutes without sugar as a control. The allergenic potential of tested extract was examined using the basophil activation test (BAT) and inhibition ELISA. Maillard reaction products (MRP) were identified by western blot.

## Results

Patient-dependent differences in the levels of activated basophils were seen in BAT test. When compared to raw soy an increase (from 2 to 5 times; 2 patients) or decrease (from 2 to 36 times; 3 patients) of allergenicity for all processed extracts was seen. For 2 patients miscellaneous changes for different processing conditions were observed. For 70% of the patients SPE heated for 30 minutes with glucose stimulated basophils stronger than control SPE. The neo-allergens formed during heating of SPE with glucose were characterized on blots as high-molecular weight agglomerates of proteins and sugars, which contain advanced MRP. Their inhibitory ability for specific IgE binding was established in ELISA.

## Conclusion

Maillard-type neoallergens formed during soy processing can trigger stronger basophil stimulation than raw soy proteins in soy allergic patients. The use of only raw soy extracts in diagnostic tests may provide false negative results.

